# Feasibility of using contrast-free quantitative magnetic resonance imaging for liver sparing stereotactic ablative body radiotherapy

**DOI:** 10.1016/j.ctro.2024.100859

**Published:** 2024-09-13

**Authors:** Frank Brewster, Zoe Middleton, Alan McWilliam, Andrew Brocklehurst, Ganesh Radhakrishna, Robert Chuter

**Affiliations:** a Christie Medical Physics and Engineering, The Christie NHS Foundation Trust, Manchester, UK; b Leeds Teaching Hospitals NHS Trust, Leeds, UK; c Department of Radiotherapy Related Research, Division of Clinical Cancer Science, School of Medical Sciences, Faculty of Biology, Medicine and Health, The University of Manchester, UK; d Department of Clinical Oncology, The Christie NHS Foundation Trust, Manchester, UK

**Keywords:** Biologically-guided radiotherapy, Liver cancer, Functional MR, Quantitative imaging, SABR, Liver sparing

## Abstract

**Background and purpose:**

Tumours in the liver often develop on a background of liver cirrhosis and impaired liver function. As a result, radiotherapy treatments are limited by radiation-induced liver disease, parameterised by the liver mean dose (LMD). Liver function is highly heterogeneous, especially in liver cancer, but the use of LMD does not take this into account. One possible way to improve liver treatments is to use quantitative imaging techniques to assess liver health and prioritise the sparing of healthy liver tissue.

**Materials and methods:**

Anatomical T2 and quantitative iron-corrected T1 (cT1) images were made available for 10 patients with liver metastases. Functional liver volumes were automatically segmented on the quantitative images using a threshold. Liver stereotactic ablative body radiotherapy (SABR) plans were made using a departmental protocol. Liver-sparing plans were then made by reducing the dose to the functional sub-volume.

**Results:**

The sparing plans achieved a statistically significant (p=0.002) reduction in the functional liver mean dose, with a mean reduction of 1.4 Gy. The LMD was also significantly different (p=0.002) but had a smaller magnitude with a mean reduction of 0.7 Gy. There were some differences in the planning target volume D_99%_ (p=0.04) but the sparing plans remained within the optimal tolerance and the D_95%_ was not significantly different (p=0.2).

**Conclusions:**

This study has, for the first time, demonstrated the use of cT1 maps in radiotherapy showing significant reductions in dose to the healthy liver. Further work is needed to validate this in liver cancer patients, who would likely benefit most.

## Highlights

• Current liver SABR techniques do not account for heterogenous liver function.
• Contrast-free iron-correct T1 MR sequences can be used to image liver function.
• Liver SABR plans were made to prioritise sparing of healthy liver on an MR-Linac.
• Sparing plans achieved a 1.4 Gy reduction of the functional liver mean dose.

## Graphical abstract

**Figure:**
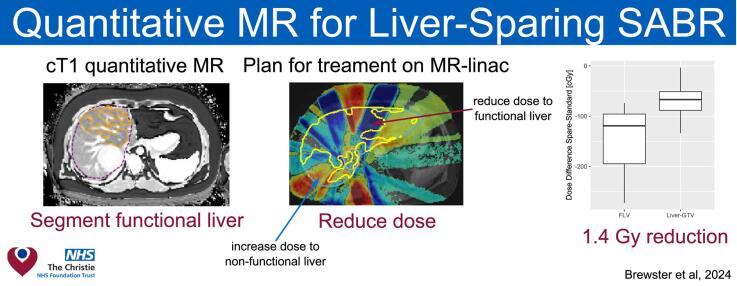

## 1 Introduction

Malignant cancerous lesions in the liver can arise from metastatic or primary disease with metastases being significantly more common [1]. The liver is a common site for metastases for several primary sites owing to its dual blood supply but colorectal cancer (CLC) is the most common primary with approximately 25–30 % of patients developing liver metastases [2]. CLC is the second most common cause of cancer mortality worldwide representing approximately 1 in 10 cases and deaths [3].

Hepatocellular carcinoma (HCC) is the most common form of primary liver cancer (PLC), accounting for approximately 51 % of PLC diagnoses in the UK [4]. The incidence of HCC has increased significantly in recent years with the number of diagnoses trebling between 1997 and 2017 [5]. HCC is a largely preventable cancer and while survival rates have improved, they remain poor with 46.7 % of patients alive after 1 year and only 17.3 % alive after 5 years in the UK. As a result, there is a desire to find new or improved low-cost treatments to improve survival and radical treatment rates.

Stereotactic ablative body radiotherapy (SABR) is an effective locoregional treatment for both unresectable early HCC, with a local control rate of 68–95 % at 3-years [6] and metastatic colorectal cancer, with a local control rate of 60–90 % at 2 years [7]. In addition, SABR may have advantages over other locoregional therapies in terms of local control, progression-free survival and toxicity [8]. However, the use of SABR in the liver is hampered by high levels of toxicity, in particular, radiation-induced liver disease (RILD) [9]. HCC patients are particularly susceptible to RILD, as HCC is often developed on a background of cirrhosis and impaired liver function [10]. As a result, access to SABR for both cohorts is limited by liver function according to a patient’s Child-Pugh score with the NHS in England only commissioning liver SABR for patients with a Child-Pugh score ≤ A6 [11], [12].

Current methods to avoid RILD involve limiting the liver mean dose (LMD) [13]. The dose volume analyses used to derive these tolerances assume homogenous liver function; however, liver function is highly heterogenous, especially in HCC [14]. Quantitative imaging of liver health and its incorporation into radiotherapy planning offers the opportunity to image this heterogeneity and hence reduce the incidence of RILD and allow more patients to access this treatment.

Iron-corrected T1 (cT1) is a proprietary quantitative multiparametric magnetic resonance (qMR) imaging technique produced by Perspectum (Oxford, UK). By correcting T1 mapping sequences, cT1 quantitatively measures tissue’s free water content, and hence primarily shows inflammation. While not a direct marker of liver function, association with inflammatory processes makes cT1 a useful surrogate marker of hepatocyte function. In addition, because cT1 exploits the paramagnetic properties of water molecules, no contrast agent is required.

Previous studies looking at cT1, especially in the context of metabolic-associated fatty liver disease and its severe form, non-alcoholic steatohepatitis (NASH), have demonstrated its correlation with histopathological features common to both NASH and RILD [15], [16]. Elevated cT1 levels have been associated with adverse liver and cardiac outcomes [17]. Moreover, an application of cT1 in patients with liver metastases (n = 136) or PLC (n = 7) showed that higher cT1 values were linked to longer hospital stays and reduced liver performance post-resection [18]. These research findings suggest a potential role of cT1 to identify functioning liver tissue but to date have been primarily conducted in a non-oncology setting and none of them investigate radiotherapy patients.

The introduction of hybrid treatment platforms like the MR-linac (MRL)[19] offers the opportunity to image radiotherapy patients on treatment without additional imaging sessions. The lack of contrast-agent involved in cT1, its established use in the clinical in surgical practice and FDA approval uniquely positions the technique for use on an MRL for longitudinal imaging for treatment response monitoring or bioadaptation. This retrospective feasibility study investigates the use of cT1 in RT for the first time. We aimed to evaluate if cT1 maps could be used to produce regions of interest (ROIs) identifying functional liver and hence if there is optimisation space to reduce dose to the function regions without increasing doses to other organs.

## 2 Materials and methods

This study conducted a comparison between liver SABR treatment plans integrating cT1 maps for assessment of liver function and those created using the standard of care. This comparative analysis was designed to evaluate both the feasibility of acquiring usable ROIs for treatment planning from cT1 maps and the advantages of incorporating these into the plan optimisation. Specifically, we aimed to demonstrate possible reductions in dose to functional liver tissues without detriment to the overall plan quality or increasing dose to healthy organs.

The analysis involved a retrospective evaluation of ten patients enrolled in the Precision 1 trial (clinicaltrials.gov ID NCT04597710). These consisted of both a cT1 qMR image and a T2 anatomical image for treatment planning. All patients originally had colorectal cancer with liver metastases planned to undergo surgical resection and were selected by the Precision 1 study team to represent a suitable range of tumour sizes localised within the liver [20]. The sample size was selected to be proportional to the aims of the study (feasibility).

The cT1 maps were generated using LiverMultiscan (Perspectum Ltd, UK). A T1 acquisition was performed using the MOLLI-T1 method (previously described [21]) using the following parameters: TR=4.77 ms, TE=1.97 ms, flip angle = 35°, acquired matrix 192x144, with GRAPPA acceleration of 2 with 24, slice thickness 8 mm, field-of-view 440 mm, phase field-of-view 75 %. T2* acquisition for iron-correction used 2D multiple gradient echo acquisitions with 6 echo times.

### 2.1 Segmentation of the functional volume

To produce more clinical useful sparing volumes, the images were first downsampled by a factor of two in the left–right and anterior-posterior directions (from 1.1 mm to 2.2 mm). Images were then cropped to the liver ROI from the T2 image. As both images were acquired in the same session, the registration offset was assumed to be negligible. This was validated visually by comparison with the liver contour on the anatomical image.

**Fig. 1:**
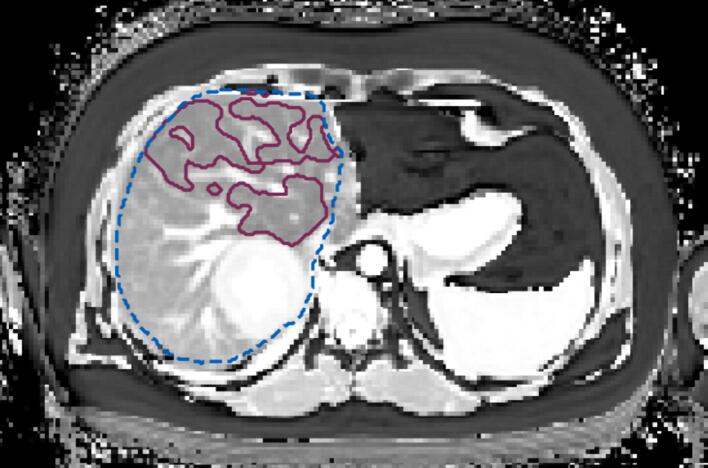
An example of a segmentation of the function liver sparing contour (maroon solid) with the liver from the anatomical image (dashed blue). (For interpretation of the references to colour in this figure legend, the reader is referred to the web version of this article.)

To create a functional liver volume (FLV), the cT1 map was then thresholded, with a value < 800 ms considered functional. This value was chosen based on literature [15], [22], [23]. The masks were smoothed with a recursive Gaussian filter (σ=4) and thresholded by the median pixel value to remove smaller regions. The value of σ was optimised visually to provide usable sparing volumes. This was done to prevent the optimiser overfitting and producing overly complex plans for no clinical gain. An example of the resulting FLV is shown in Fig. 1.

### 2.2 Treatment planning

Organs at risk (OARs) and the target were contoured as appropriate on the T2 image including the gross tumour volume (GTV), liver (including the hilus and great vessels), spinal canal, chest wall, duodenum, small and large bowel, oesophagus, heart, and kidneys. The GTV was contoured by a clinical oncologist and OARs were contoured either by a clinical oncologist or an appropriately trained physicist according to the NRG-GI003 trial protocol (clinicaltrials.gov ID NCT03186898).

To enable dose calculation, bulk density overrides were applied to the T2 MR. Population-based relative (to water) electron density (rED) values were determined based on patients previously treated or planned for the MRL. The lungs were overridden to 0.328 rED based on six previously treated lung patients. The vertebrae were overridden to 1.153 rED and the remaining tissue to 0.988 rED, based on six previously treated pancreas patients and four patients used in the development of liver treatments (see Appendix A).

Plans were made using an Elekta Unity MRL beam model on Monaco (v5.51.11, Elekta AB, Stockholm, Sweden) with twelve equispaced intensity-modulated RT (IMRT) beams between gantry angles of 168° and 24° to spare contralateral OARs. The plans were made according to the departmental class solution for the five-fraction photon arm of NRG-GI003. Although this protocol is designed for HCC patients, the same prescription is used in metastatic disease. The protocol was chosen as it uses tighter LMD tolerances than our departmental protocols for HCC or metastases and therefore represents a worst-case scenario. The prescription dose covering 95 % of the PTV was limited by the LMD, as shown in Table B1. Dosimetric criteria were adapted from NRG-GI003 to meet local clinical practice, as shown in Table B2. The planning target volume (PTV) was formed by a 5 mm expansion from the GTV. The departmental protocol is for a motion-adapted GTV and an internal target volume (ITV) to be formed from the GTV and a 4D computed tomography (4DCT) scan; however, no respiratory information was available for these patients and so the PTV was formed directly from the GTV instead. Planning risk volumes (PRVs) were expanded from critical OARs using a margin of 5 mm to account for motion. For four of the patients, the PTV was contracted from gastrointestinal OARs to avoid these being over the mandatory tolerance.

Once a plan using the standard solution had been made, an additional “parallel” planning objective was added to the FLV to reduce dose in this region. This objective is designed to model the radiobiological behaviour of a parallel organ by optimising equally across the dose range, rather than just the level set. In practise, this allows the optimiser to reduce low dose to a large volume or high dose to a small volume for the same gain. This constraint was reduced until further reductions compromised PTV coverage below the optimal tolerance. No other changes were plan to the sparing plans.

The dosimetric criteria in Table B2 as well as the Liver-GTV mean dose were extracted for all plans and paired Wilcoxon signed-rank tests were used to test for significant differences (α=0.05) between the standard and sparing plans. Plans were also reviewed by a clinical oncologist and a second physicist.

## 3 Results

### 3.1 Segmentation

The size of the FLV produced varied greatly between patients, with some patients showing as having highly functional livers in the scan. Table 1 shows the volumes for the FLV as well as the volume of liver inside the cT1 map field of view (FOV), and the proportion of this segmented as functional, in the range of 17–83 % (Ratio).

**Table 1:** Table of information on liver volumes and segmentations showing volumes of the whole liver (Liver), gross target volume (GTV), liver with GTV subtracted used for prescription setting (Liver-GTV), liver inside the functional image field of view (FOV) and functional liver volume (FLV). The ratio of functional liver to total liver available to be segmented is also shown (Ratio) as well as the prescription value used (Rx).

*Patient*	*Liver [cc]*	*GTV [cc]*	*Liver-GTV [cc]*	*FOV [cc]*	*FLV [cc]*	*Ratio*	*Rx [Gy]*
*1*	1593	12	1581	773	276	36 %	50
*2*	1353	11	1341	855	662	77 %	50
*3*	1379	92	1287	724	115	16 %	40
*4*	1320	3	1317	880	730	83 %	50
*5*	1992	309	1687	990	654	66 %	40
*6*	1842	12	1830	970	823	85 %	50
*7*	1873	10	1863	799	138	17 %	50
*8*	1985	45	1940	915	689	75 %	50
*9*	1385	40	1346	697	498	71 %	50
*10*	1906	32	1874	842	476	56 %	50

### 3.2 Treatment plans

**Fig. 2:**
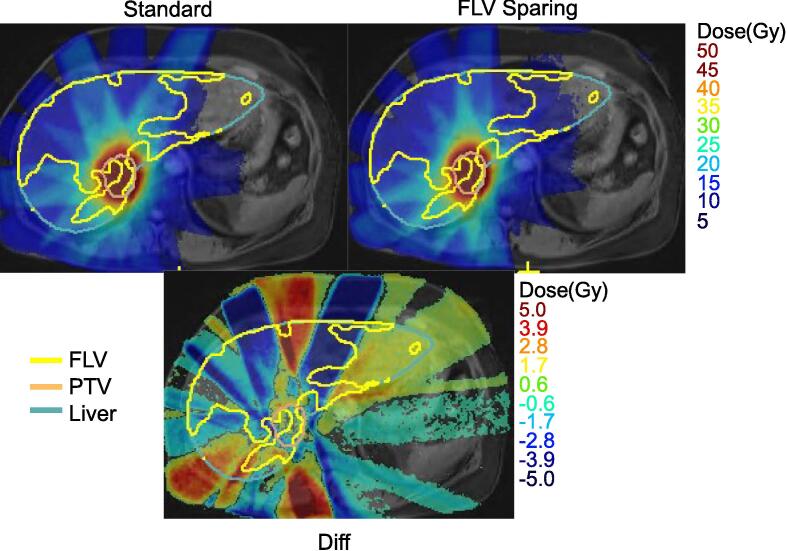
Dose maps showing the standard plan (top left) and functional liver volume (FLV) sparing plan (top right) as well as the FLV (yellow), the PTV (beige) and the liver (blue). The bottom image shows the subtraction of the standard plan from the sparing plan with positive values indicating a hotter dose on the sparing plan. (For interpretation of the references to colour in this figure legend, the reader is referred to the web version of this article.)

Clinically acceptable plans could be made for all patients. An example of functional sparing is shown in Fig. 2, showing the reduction in dose through the anterior of the liver and a lateral increase without significant changes in PTV dose. Eight of the patients could be planned to 50 Gy, but two had to be reduced to 40 Gy (as per Table B1). These were the patients with the largest GTV volumes of 92 and 309 cc for patients 3 and 5 respectively, as shown in Table 1. Both the standard and sparing plans were deemed clinically acceptable for all patients and all dosimetric criteria were inside mandatory tolerances.

The mean dose to the functional subvolume was found to be significantly different between the standard and sparing plans (p=0.0020), as shown in Table 2. The Liver-GTV mean dose difference was also significant (p=0.0020) and the difference in D_99%_ for the PTV was borderline significant (p=0.037). There were no significant differences in any other metrics detected, indicating that sparing plans had not simply reduced the total plan dose or increased dose to other OARs, as seen in Table 2 (also shown in Fig. C1).

**Table 2:** Table of median and upper and lower quartiles as well as p-values for paired Wilcoxon signed-rank tests between the standard and sparing plans where FLV is the functional liver volume, PRV indicates a planning risk volume and GTV is the gross tumour volume. p-values below the significance level are shown in bold.

*Structure*	*Quantity*	*Median (IQR)*		p*-value*
		*Standard*	*Sparing*	
*PTV*	D_95%_	103.2 % (101.2 %, 105.3 %)	103.1 % (101 %, 104 %)	0.1934
	D_99%_	**100.4 % (98.5 %, 102.7 %)**	**99.9 % (97 %, 101.5 %)**	**0.0371**
	D_0.03cc_	119.2 % (118.1 %, 124.4 %)	121.6 % (119.4 %, 125.9 %)	0.1055
*Spinal Cord PRV*	D_0.5cc_	10.25 (8.72, 16.15) Gy	10.51 (938, 15.59) Gy	0.8457
*Stomach PRV*	D_0.5cc_	10.15 (8.14, 11.96) Gy	10.94 (6.56, 13.65) Gy	0.4922
*Duodenum PRV*	D_0.5cc_	3.86 (1.58, 7.21) Gy	2.7 (1.57, 7.08) Gy	0.3223
*Small Bowel PRV*	D_0.5cc_	1.07 (0.71, 4.01) Gy	1.12 (0.7, 3.59) Gy	0.5566
*Large Bowel PRV*	D_0.5cc_	11.17 (3.8, 24.8) Gy	11.19 (3.9, 23.94) Gy	0.3008
*Oesophagus PRV*	D_0.5cc_	9.63 (7.97, 15.98) Gy	9.27 (8.21, 17.9) Gy	0.2324
*Kidneys*	D_33%_	0.8 (0.7, 1.22) Gy	0.81 (0.69, 1.23) Gy	0.7211
	Mean	1.04 (0.68, 1.66) Gy	1.04 (0.68, 1.72) Gy	0.7695
*Heart PRV*	D_0.5cc_	6.47 (4.72, 10.25) Gy	5.61 (4.41, 8.31) Gy	0.6250
*FLV*	Mean	**12.81 (10.37, 15.02) Gy**	**11.29 (9.06, 13.76) Gy**	**0.0020**
*Liver-GTV*	Mean	**9.56 (8.44, 10.89) Gy**	**8.57 (7.89, 10.17) Gy**	**0.0020**

**Fig. 3:**
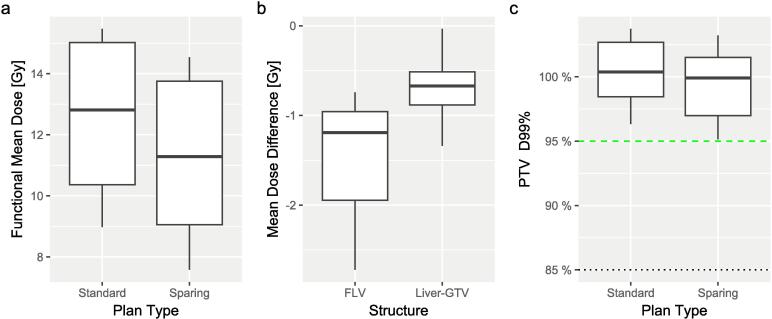
Box and whisker plots showing (a) the mean dose to the functional liver for both the standard and liver-sparing plans, (b) the mean dose differences (spare-standard) in Gy between the sparing and standard plans for both the function liver volume (FLV) and the whole liver without the gross tumour volume (GTV). This shows smaller reductions for the liver compared with the FLV, (c) the dose received by 99% of the planning target volume (PTV) for the standard and sparing plans. The optimal tolerance is shown by the green dashed line and the mandatory tolerance by the black dotted line. (For interpretation of the references to colour in this figure legend, the reader is referred to the web version of this article.)

The sparing plans were able to achieve a mean reduction in mean dose to the FLV of 1.4 Gy, as shown in Fig. 3a. The Liver-GTV mean dose was also reduced but to a lesser extent than the reductions in FLV with a mean of 0.7 Gy, as shown in Fig. 3b. Although the PTV D_99%_ reductions were significant, none of these were below the optimal tolerance, as shown in Fig. 3c and so were not considered dosimetrically or clinically significant. To ensure that changes in PTV coverage were not the driving factor in the FLV dose reductions, the standard plans were rescaled to have the same PTV D_95%_ as the sparing plans. The statistical significance of the differences in FLV dose remained (same p-value).

The V_10Gy_ for the liver was also extracted and had statistically significant differences between the plans (p=0.002) with all plans remaining above the UK SABR consortium optimal tolerance of 70 % [24], however; the fact that the plans were not optimised at this dose level limits the applicability of this result.

During planning it was generally seen that it was more difficult to achieve reductions in dose to the FLV for patients with larger FLVs. On investigation, there was little correlation seen ($R^{2}=0.1$) between the percentage FLV (compared to the volume of liver inside the qMR FOV) and the FLV dose reduction.

## 4 Discussion

In this retrospective planning study, cT1 maps have been used for the first time in the planning of liver SABR. A statistically significant reduction in dose to the functional region was achieved without a clinically significant change in overall plan quality. Although there were reductions in PTV coverage, the plans were still within optimal tolerances and statistically significant reductions were only seen in the D_99%_ and not in the D_95%_.

The utility of biological information in HCC SABR has already been shown using indocyanine green clearance blood tests of global liver function [25]. Feng et al carried out a phase 2 trial of 90 patients with PLC or metastases, planning SABR doses to maintain liver function based on mid-treatment liver function [26]. They reported lower complication rates than expected as well as 99 % 1-year local control. Jackson et al performed a similar study with 80 HCC patients who would normally be excluded from SABR (Child-Pugh score B) and showed the approach allowed for 92 % 1-year local control and acceptable toxicity [27]. These trials show that there is clearly room for toxicity reduction using biological information in these treatments but do not take into account the highly heterogeneous nature of liver function. This could allow for improved planning and hence lower rates of toxicity.

Several studies have investigated the use of quantitative imaging for liver RT. The majority of existing literature explores the use of ^99m^Tc and single photon emission computed tomography (SPECT) to adapt SABR plans and the feasibility of this from a planning perspective has been well established [28], [29]. While these studies show promise for reducing the incidence of RILD, the ionising nature of the modalities used restricts longitudinal imaging and prevents daily on-treatment imaging. In addition, prospective studies carrying higher risk are more difficult to justify while the clinical benefit is not fully known.

MR imaging offers the opportunity to acquire quantitative imaging without ionising radiation and the feasibility of using qMR for FLV avoidance has been investigated in other studies. The use of DCE-MR with gadolinium (Gd) based contrast agents has been shown to correlate with established liver function tests [30]. Tsegmed et al carried out a planning study with 20 HCC patients using DCE-MR to define a functional liver avoidance region [31]. They were able to achieve a mean reduction to the FLV mean dose of 0.5 Gy, without significant changes to the plan quality. DCE-MR scans image perfusion and although this correlates well with function, Simeth et al demonstrated that mismatches between perfusion and function resulted in only 64.9 % mean agreement between the two [32]. In the worst case, this resulted in a 10 % difference in FLV mean dose. Although these studies show DCE-MR as a potential modality for treatment planning, repeated administration of Gd-based contrast agents carries safety concerns due to the build-up of Gd in the brain [33]. This prevents longitudinal imaging studies, response assessment and hampers efforts to verify the repeatability of the technique, a known weakness of qMR quantitative imaging biomarkers (QIBs).

Hybrid treatment platforms, such as MRLs, give the opportunity to acquire QIBs while patients are on treatment at little or no cost to treatment times. Lee et al have developed a technique to segment functional liver on an MRL using super-paramagnetic iron oxide nanoparticles (SPION) as a contrast agent on R2*-MR [34]. They reported successful segmentation on 12 patients with a mix of PLC and metastases but are yet to incorporate these into treatment plans. The expense and regulatory difficulties associated with SPION may make the technique more difficult to implement on a larger scale [35] so more work is required to validate the feasibility of the technique. The cT1 sequences used in this study do not require any contrast agent, removing concerns about toxicity and cost. This makes them much better suited for use on an MRL as liver function could be measured on each treatment fraction. These could then be used for treatment response monitoring, to identify patients at risk of RILD and deescalate dose mid-treatment. It is also possible that spatial changes in liver function during RT could be measured and used to adapt the treatment plans to take this into account.

The use of cT1 images to map liver function has not yet been explored in an RT context. This study represents the first introduction of this technique for liver SABR planning. Whilst cT1 has been linked with clinical outcomes in patients with NASH [15], [36] and patients undergoing resections for liver metastases [18], there has not yet been any validation in RT patients and very little in those with HCC. This represents a significant hurdle in the translation of this technique. Before any intervention is made, cT1 would need to be linked with established clinical factors influencing the risk of RILD. The repeatability and reproducibility would also need to be established as the biomarker has currently not been used longitudinally.

Although efforts were made to select representative cases of both CRC and HCC, the diagnoses of the patients used may influence the findings of this study. CRC patients are likely to have superior liver function to HCC patients, making the sparing both more difficult to achieve and less clinically relevant. The incidence of cirrhosis and hence the proportion of remaining FLV is likely to be lower in PLC patients in general but especially in HCC and so reduction in function for CRC patients are more likely due to chemotherapy. For this reason, a planning solution was chosen that represented HCC planning as well CRC with a view to validate this result in an HCC SABR patient group in future. This feasibility study provides the motivation to carry out such work.

The value of 800 ms used to threshold the FLVs was chosen based on discussion with the manufacturer as well as available literature [15], [16], [22], [23], [36]. The exact value used in previous studies varies and so the use of 800 ms represents a pragmatic choice for the purposes of this feasibility study as the CRC patient population does not represent the ideal target patient population. This clearly represents a limitation and this value needs defining further with links to patient outcomes before the method is properly established. The value was chosen based on surgical cohorts in which cT1 is summarised per-lobe to guide surgical removal of the tumour while leaving the healthiest lobes in-tact. The transfer to RT needs further consideration as we can account for cT1 spatially during optimisation. This also means that the threshold value carries more importance in this context and so needs to be better optimised. Alternatively, the signal could be used as a function for dose reduction in which the lowest functioning voxels would have less weight during optimisation. This will form future work as part of a prospective data collection study which will capture signal from the full liver volume.

Another limitation of using images from a sperate study is their field of view. The cT1 images were acquired with the purpose of calculating a future liver remnant volume for surgery and although they cover the extent of disease, they do not cover the entire liver. This reduces the possible FLV but this was judged to be adequate for the purposes of this study as fall-off in the superior-inferior direction tends to be steeper.

Dose calculation was enabled on T2 MR images in this study using population-based bulk density overrides. Although this introduced uncertainties, these have been shown to be minimal [37] and this technique is used locally in routine clinical practise on the MRL. In addition, this study primarily performs comparisons between plans calculated using the same method and the use of an alternative synthetic-CT method could introduce additional uncertainty.

While the LMD showed an equally significant reduction in dose to the FLV, the magnitude of this reduction was lower with a mean of 0.7 Gy compared to 1.4 Gy. Considering that most (7/10) of the patients had an FLV covering more than 50 % of the liver within the cT1 FOV, reductions in LMD are unavoidable and the smaller magnitude shows that these same reductions could not be achieved by reducing the LMD alone.

There were some small differences in PTV coverage between the standard and sparing plans. To ensure that this was not the driving factor in the FLV dose reductions, the standard plans were rescaled to have the same PTV D_95%_ as the sparing plans; however, these results are not shown as the differences in PTV coverage are very small and the dose was calculated using a Monte-Carlo algorithm with a 1 % uncertainty (as per departmental protocol) which most of the plans fall well within; all plans remained within optimal tolerances and so demonstrate that there is optimisation space to reduce FLV dose whilst remaining within dosimetric criteria.

The departmental protocol for liver SABR uses a 5 mm ITV to PTV margin and uses 4DCT to create an ITV. No respiratory information was available for the patients in this study. Although it would be possible to approximate the magnitude of respiratory motion using population data, respiration is highly variable both between patients and between cohorts [38]. Instead, the same PTV margin was used on the GTV. Implementing this approach would require treating using respiratory gating. This technique is already in use in some centres and will soon be available on the Elekta Unity MR-linac platform that was used for this planning study. Considering the risk of RILD for HCC patients, it is likely that these patients would be treated using gating if this was available as removing respiratory motion has been shown to reduce LMD and allow for higher prescriptions [39].

The plans in our study showed a mean 1.4 Gy reduction in FLV mean dose. This difference could be clinically significant for some patients, especially those with the most impaired liver function. A systematic review by Zhou et al found that functional avoidance planning studies reduced the mean dose to FLV by a mean of 1.0 Gy and these studies reported a low incidence of RILD [28]. There was also some correlation seen between the FLV percentage and the achievable reduction in FLV mean dose, although this was weak. This suggests that larger reductions could be possible for HCC patients.

A strength of this study is the potential translatability of the imaging sequences. Their clinical use has already been established in a surgical setting but this is the first study to apply them to RT. The lack of contrast agent negates toxicity concerns associated with Gd and reduces cost and scan time. On a hybrid platform, it means that the scans could be run in parallel with treatment at no additional cost. The ability to scan daily opens the opportunity to more closely monitor and adapt to treatment response. It may also mean that it is easier to verify the reproducibility of the QIB, both in patients and healthy volunteers.

## 5 Conclusion

This study has demonstrated the feasibility of creating FLVs using a contrast-free qMR imaging sequence and that the volumes produced can be used in liver SABR planning to produce a mean FLV dose reduction of 1.4 Gy. All sparing plans produced were judged to be clinically acceptable and could produce a meaningful reduction in RILD, potentially allowing more patients to access this treatment. Further work is required to verify these results in a more relevant patient cohort and to assess the repeatability of the QIB.

## Declaration of competing interest

The authors declare that they have no known competing financial interests or personal relationships that could have appeared to influence the work reported in this paper.

## Figures and Tables

**Table:** 

*Type*	*rED*	
	*Vertebra*	*Body*
*Treated*	1.155	0.953
	1.141	0.998
	1.140	0.977
	1.149	1.004
	1.158	0.949
	1.138	1.017
*Evaluation*	1.168	1.005
	1.181	0.968
	1.140	1.016
	1.164	0.991
*Mean*	1.153	0.988

**Table:** 

*Type*	*Lung-PTV rED*
*Treated*	0.371
	0.398
	0.368
	0.217
	0.306
	0.310
*Mean*	0.328

**Table:** 

*Prescription Dose*	*Liver-GTV Mean Dose*
50 Gy	<13 Gy
45 Gy	<14 Gy
40 Gy	<15 Gy
35 Gy	<15.5 Gy
30 Gy	<16 Gy

**Table:** 

*Structure*	*Quantity*		*Optimal*	*Mandatory*
*PTV*	D_95%_	≥	95 %	90 %
	D_99%_	≥	95 %	85 %
	D_0.03cc_	≤	120 %	150 %
*Spinal Cord PRV*	D_0.5cc_	<	25 Gy	28 Gy
*Stomach PRV*	D_0.5cc_	<	30 Gy	32 Gy
*Duodenum PRV*	D_0.5cc_	<	30 Gy	32 Gy
*Small Bowel PRV*	D_0.5cc_	<	30 Gy	32 Gy
*Large Bowel PRV*	D_0.5cc_	<	32 Gy	34 Gy
*Oesophagus PRV*	D_0.5cc_	<	30 Gy	34 Gy
*Kidneys*	D_33%_	<	15 Gy	17 Gy
	Mean	<	10 Gy	12 Gy
*Heart PRV*	D_0.5cc_	<	22 Gy	−
*Chest Wall*	D_2cc_	<	35 Gy	40 Gy

## Appendix A . Electron density information

Table A1. Relative (to water) electron density (rED) values for 10 pancreas patients for the vertebrae and body. Patients had either been previously treated on our MR-linac or used in the commissioning of pancreas SABR. Their means were used for the patients in this study.Table A2. Relative (to water) electron density (rED) values for 6 previously treated lung patients for the lung with the planning target volume (PTV) subtracted. Their mean was used for the patients in this study.

## Appendix B. Dosimetric criteria

Table B1. Maximum allowed mean doses for the liver with the gross tumour volume (GTV) subtracted for each prescription dose. The prescription dose was reduced if the constraint could not be met.Table B2. Dosimetric criteria used when making the plans where PTV is the planning target volume, PTV_IMRT is the PTV cropped from critical organs and PRV is the planning risk volume to account for motion. Percentage values are relative to the prescription dose.

## Appendix C . Full dosimetric results

Fig. C1. Box and whisker plot of all absolute dosimetric criteria for both plans types where planning risk volumes (PRVs) are grown via a 0.5 cm expansion. Dosimetric statistics have their usual meaning and LMD and FLV refer to the liver mean dose and function liver volume respectively.

Fig. C2. Box and whisker plot of all absolute dosimetric criteria for both plans types. Dosimetric statistics have their usual meaning, PTV refers to the planning target volume and Rx refers to the prescription dose.
